# Use of Hypertonic Continuous Venovenous Hemodiafiltration to Control Intracranial Hypertension in an End-Stage Renal Disease Patient

**DOI:** 10.4061/2010/391656

**Published:** 2010-07-15

**Authors:** Stephen I. Rifkin, Ali R. Malek, Reza Behrouz

**Affiliations:** ^1^Division of Nephrology, Department of Internal Medicine, University of South Florida College of Medicine, Tampa, 33612, USA; ^2^Interventional Neurology Program, Comprehensive Stroke Center, St. Mary's Medical Center, West Palm Beach, 33407, USA; ^3^Section for Neurological Critical Care, Department of Neurology, University of South Florida College of Medicine, Tampa, 33612, USA

## Abstract

Continuous venovenous hemodiafiltration (CVVHDF) using solutions designed to maintain hypernatremia is described in an end-stage renal disease (ESRD) patient with cerebral edema (CE) due to an intracerebral hemorrhage (ICH). Hypernatremia was readily achieved and maintained without complication. CVVHDF should be considered as an alternative treatment option in ESRD patients with cerebral edema who require hypertonic saline therapy.

## 1. Introduction

Osmotherapy is an option for the treatment of cerebral edema due to acute brain injury. It is used to prevent progression of CE and lower intracerebral pressure (ICP). Mannitol and hypertonic saline (HTS) are the drugs currently available for this purpose [1]. Mannitol would be inappropriate in a patient with end-stage renal disease (ESRD) due to its accumulation in a patient without excretory function. Hypertonic saline would be acceptable only as long as its accumulation and the excess extracellular volume generated by the resulting hypertonicity could be controlled. A patient with ESRD is described who developed an ICH with subsequent CE and was placed on continuous venovenous hemodiafiltration (CVVHDF) using fluids specially designed to maintain a hypertonic state with goal serum sodium of approximately 150–155 meq/L. 

## 2. Case Report

The patient is a 45-year-old male admitted after developing a headache, slurred speech, and left hemiparesis during his routine hemodialysis treatment. A CT scan of the brain showed a 4.4 × 2.5 cm area of hyperattenuation in the right basal ganglia area, moderate edema associated with the ICH, and mass effect with a 2 mm right-to-left midline shift. His admission serum sodium was 139 meq/L. He had been on hemodialysis for the past 7 to 8 years. He was known to be hepatitis C antibody and hepatitis B antigen positive. He also had a history of hypertension and asthma. The day after admission a repeat CT scan demonstrated that the edema and mass effect had increased and that the midline shift had increased to 10 mm. Because of compression on the third ventricle, mild hydrocephalus had also developed. A ventriculostomy catheter was inserted the day after admission. That same morning, he was started on continuous venovenous hemodiafiltration (CVVHDF) using regional citrate anticoagulation. Our standard CVVHDF protocol is based on that of Mehta et al. [2] but uses a fixed rate of citrate. The standard protocol has a dialysate sodium of 117 meq/L and a predilution replacement fluid sodium of 135 meq/L. This was adjusted to a dialysate sodium of 127 meq/L and a predilution replacement fluid sodium of 155 meq/L. In addition, shortly after the initiation of CVVHDF, the patient was given a 250 cc bolus of 3% saline followed by an infusion at 100 cc/hr to quickly elevate his serum sodium. The infusion was discontinued the next day, and the patient was maintained on hypertonic CVVHDF. CVVHDF was continued for a total of four days. Lab values were frequently monitored. Minor adjustments to the solutions were made to keep the serum sodium near the goal of approximately 155 meq/L. Peak serum sodium was 157 meq/L. Serum sodium was successfully maintained in the goal range. Intracranial pressure and cerebral perfusion pressure were frequently monitored and were always satisfactory. Unfortunately, the patient's neurologic status did not improve. After four days of CVVHDF, the decision was made to allow the serum sodium to slowly normalize. CVVHDF was stopped, and the patient was placed back on intermittent hemodialysis. Dialysate sodium of 147 meq/L was used for one hemodialysis treatment and then 145 meq/L was used for the next treatment. The serum sodium came down to 144 meq/L. Several followup head CT scans showed no improvement. At that point, the family requested further therapy be discontinued, and the patient expired shortly thereafter.

## 3. Discussion

Osmotherapy is an effective treatment option to control CE and reduce intracranial pressure in patients with acute brain injury. Mannitol and HTS are the usual drugs used. Both can have significant side effects. Mannitol has been reported to cause a variety of electrolyte disturbances and acute renal failure [3]. In an ESRD patient who is anuric, mannitol could accumulate in the brain tissue and paradoxically increase CE. HTS causes hypernatremia and extracellular volume expansion and can cause metabolic acidosis and hypokalemia among other side effects [4]. HTS has been shown to be effective in patients resistant to mannitol therapy [4, 5]. HTS may also have a beneficial effect on mortality in the treatment of raised ICP when compared to mannitol [6]. It has been reported that serum tonicity as high as 365 mosm/l can be safely achieved with HTS therapy whereas mannitol therapy is limited to a maximum of 320 mosm/l [7]. When hypernatremia, whether or not a consequence of osmotic therapy, was evaluated in patients in a neurologic ICU, it was an independent predictor of mortality only when the peak serum sodium exceeded 160 meq/L [8].

It has recently been suggested that continuous renal replacement therapy, due to greater cardiovascular and intracranial stability, is the preferred method to treat patients with acute brain injury and renal failure [9]. We treated our patient with CVVHDF using specifically prepared solutions. Our pharmacy does not use commercially prepared solutions but rather uses an automatic compounder to custom mix the ordered solutions. Thus, it was straightforward for the pharmacy to mix the requested solutions for hypertonic CVVHDF. This simplified the patient's regimen as the need for a constant hypertonic saline drip was eliminated. We observed no untoward effects from the hypertonic dialysis, and the serum sodium was readily maintained in the goal range. We are aware of only one similar report in the literature, that of Hofmann et al. using continuous venovenous hemofiltration (CVVH) and that is only reported in abstract form [10]. We feel that hypertonic CVVHDF should be considered a reasonable treatment option for acute brain injury in patients with renal failure.

## 4. Conclusion

CVVHDF with solutions designed to induce hypernatremia was used to treat an ESRD patient with CE due to an ICH. We were able to maintain the serum sodium at goal, and no adverse effects of the procedure were noted. We suggest hypertonic CVVHDF be considered an option in the treatment of ESRD patients with CE due to acute brain injury.
